# The experience of immersive virtual reality in patients with heart failure during cardiac rehabilitation: a qualitative study

**DOI:** 10.3389/fmed.2025.1578399

**Published:** 2025-05-14

**Authors:** Valentina Micheluzzi, Gavino Casu, Francesco Burrai, Antonella Canu, Antonio Sircana, Pierluigi Merella, Angelo Laconi, Andrea Chelo, Piero Cozzula, Michela Piredda, Ercole Vellone

**Affiliations:** ^1^Department of Biomedicine and Prevention, University of Rome “Tor Vergata”, Rome, Italy; ^2^Clinical and Interventional Cardiology, University Hospital of Sassari, Sassari, Italy; ^3^Department of Medicine, Surgery and Pharmacy, University of Sassari, Sassari, Italy; ^4^Research Unit of Nursing Science, Department of Medicine and Surgery, Campus Bio-Medico of Rome University, Rome, Italy; ^5^Faculty of Nursing and Midwifery, Wroclaw Medical University, Wrocław, Poland

**Keywords:** cardiac rehabilitation, content analysis, heart failure, interviews, qualitative research, virtual reality

## Abstract

**Background:**

To alleviate symptoms and reduce adverse outcomes associated with heart failure, international guidelines strongly recommend cardiac rehabilitation. However, patient adherence to rehabilitation programs remains suboptimal, leading to unfavorable effects on key cardiac outcomes. Immersive virtual reality (iVR) is a promising intervention to improve adherence to cardiac rehabilitation and enhance clinical outcomes. This study aims to explore patients’ experiences with iVR during cardiac rehabilitation.

**Methods:**

A qualitative descriptive study was embedded within a randomized controlled two-arm trial involving twenty-two patients who were referred to undergo eight cardiac rehabilitation sessions, held twice a week for 30 min each. The intervention group experienced iVR in natural settings, while the control group received standard care. Semi-structured individual interviews were conducted in person after the intervention group’s eighth rehabilitation session. These interviews were audio-recorded, transcribed verbatim, and analyzed using content analysis.

**Results:**

Data from twenty-two interviews reached saturation. Content analysis generated four main categories and nine subcategories: (1) cognitive and emotional benefits of iVR (acceptability and enrichment of the rehabilitation experience, positive emotions, cognitive engagement); (2) impact on rehabilitation (physical engagement, perceived effort); (3) customizable intervention (variety of scenarios, quality and beauty of the videos); (4) negative aspects of the iVR (discomfort with the equipment, negative feelings toward the iVR devices).

**Conclusion:**

IVR is an innovative and customizable intervention that enhances the rehabilitation experience by providing cognitive, emotional, and physical benefits. It improves key aspects of rehabilitation, including time perception, motivation, enjoyment, effort perception, and anxiety management, highlighting its potential in cardiac rehabilitation. However, certain technological challenges, such as discomfort with the equipment, must be addressed to optimize the user experience.

## Introduction

Heart failure (HF) represents a significant global health challenge, affecting approximately 64 million people worldwide (1). Its prevalence, estimated at 1 to 2% in the general population of Europe and the United States, increases significantly with age, making it particularly common among older adults (1). Despite advancements in treatment, HF remains associated with high rates of hospitalization, morbidity, mortality, and disability, significantly impacting patients’ quality of life and imposing a substantial economic burden on healthcare systems (2). Globally, HF-related healthcare and societal costs are estimated at $108 billion annually, underscoring the urgent need for effective management strategies (2, 3).

Patients with HF often experience a heavy symptom burden that severely impacts both their physical and mental quality of life (4). Addressing this challenge requires implementing comprehensive strategies to manage symptoms and slow disease progression (4). Among these strategies, cardiac rehabilitation (CR) has emerged as a cornerstone of care. International guidelines strongly recommend CR (Class 1, Level A) for all HF patients who are able, in order to improve exercise capacity, quality of life, and reduce HF hospitalization, further highlighting its crucial role in HF management in secondary prevention (1).

However, adherence to CR programs remains suboptimal. Several factors, including intrapersonal barriers, clinical complexities, social determinants, and insufficient referrals by healthcare providers, contribute to this challenge (5). These limitations highlight the ongoing need to develop and implement innovative, multidisciplinary, and patient-centered strategies that can enhance CR participation among HF patients and improve their overall quality of life (5).

Immersive Virtual Reality (iVR) is an innovative and promising approach to enhancing adherence to rehabilitation programs (6–8). By providing high-quality three-dimensional (3D) images through an audiovisual head-mounted headset and controllers that enable full interaction with the virtual world, iVR users are fully immersed in realistic, multisensory environments that engage multiple senses and isolate perceptual channels from the external world, creating an illusion of reality (9–11). This deeply interactive experience has been shown to enhance adherence to rehabilitation in various fields, including musculoskeletal and neurological rehabilitation, as well as mobility and balance training, by fostering both psychological and physical responses (12–15).

A recent situation-specific theory provides a detailed guide for understanding the mechanism of iVR in rehabilitation (8). It suggests that iVR influences both proximal neurological mediators by creating a profound multisensory experience that stimulates sight, hearing, and touch, as well as distal mediators such as attention, enjoyment, and the sense of presence leading to psychological responses such as increased motivation, self-efficacy, and improved mood, as well as physical benefits like pain management and enhanced muscle strength. These effects collectively contribute to improved adherence to rehabilitation programs. The theory also identifies key moderators, such as age, gender, social support, trust in technology, and cognitive status, that influence the strength and direction of these mechanisms (8).

The impact of iVR on physical and psychological symptoms in CR is still underexplored, as most studies have focused on semi-immersive virtual reality (sVR) (16–19), a similar technology that does not ensure complete isolation from the patient’s environment, a crucial aspect for activating the mediators that lead to actual improvements in outcomes (8). Furthermore, the experiences of patients using iVR during CR, as well as in other rehabilitative contexts, remain largely unexplored. Qualitative approaches may provide deeper insights into the mind–body impact of iVR, which cannot be fully captured by simply describing its effects on physical and psychological symptoms. To address this gap, we conducted a study aimed at exploring the experiences of HF patients using iVR during CR sessions.

## Objectives

To explore the experiences of HF patients using iVR during CR.

## Methods

### Design

A qualitative study was embedded within a randomized controlled trial (RCT) designed to assess the effects of iVR on HF patients undergoing CR. According to the guidelines, the CR program in this study consisted of a total of eight 30-min treadmill sessions, supervised by healthcare personnel, twice a week for 4 weeks (20). The exercise intensity was moderate, ranging from 12 to 14 on the Borg scale (20, 21). The RCT included two groups: an intervention group that received iVR during CR and a control group that underwent standard CR without iVR. The RCT is still ongoing and evaluates various outcomes, including adherence to rehabilitation, functional capacity, perceived exertion, quality of life, heart rate, oxygen saturation, blood pressure, maximum oxygen uptake, minute ventilation/carbon dioxide production slope, oxygen pulse, and HF-related rehospitalization rates (7). As the qualitative study has reached data saturation, the findings are presented in this paper. To determine when saturation occurred, we employed a parallel analysis, continuously monitoring the data during collection to ensure that additional interviews would not yield new categories or subcategories.

### Eligibility criteria

The inclusion criteria were: (1) age > 18 years; (2) clinically stable chronic HF diagnosis; (3) left ventricular ejection fraction <40%. The exclusion criteria were: (1) conditions that contraindicate exercise training (e.g., recent fractures, severe orthopedic impairments, acute musculoskeletal injuries, or any condition compromising safe physical activity); (2) conditions that exclude the use of VR (e.g., blindness and/or deafness); (3) severe cognitive impairment, documented with a score of 0–4 on the Six-Item Screener (22, 23); (4) end-stage renal disease requiring dialysis; (5) diagnosis of advanced pulmonary diseases; (6) active malignancies; and (7) rheumatic diseases.

The qualitative strand of the study was conducted after the iVR intervention (22). The Consolidated Criteria for Reporting Qualitative Research (COREQ) checklist was used to ensure the quality of study reporting (24).

### Sample size

The first participants from the intervention group of the ongoing RCT, which has a calculated sample size of 80 participants, were interviewed. At the start of the eighth iVR session, they were provided with information about the study objectives, procedures, and methods. They were then asked if they were willing to be interviewed after completing CR sessions to share their experiences with iVR. After twenty-two interviews, parallel analysis indicated that qualitative data collection could be stopped due to data saturation (25, 26).

### Ethical considerations

The study has been approved by the Ethics Committee of the Sardinia Region (approval numbers 0531/22, 67/2024) and is being conducted in accordance with the principles of the Declaration of Helsinki (27). The study and data storage comply with the General Data Protection Regulation. A researcher approaches potential participants and obtains their voluntary, informed consent. Patient personal information is coded to ensure anonymity. The protocol is registered on ClinicalTrials.gov (identifier: NCT06115928) and is written following the SPIRIT 2013 guidelines (28).

### Procedure

The initial phase of the procedure involved assessing the feasibility of the iVR intervention and confirming that the study could be successfully conducted. All staff members were trained in the research protocol and the use of iVR technology. Sufficient funding was secured to purchase the iVR systems, and a 5G internet connection was established. The intervention had no significant environmental impact and was considered sustainable. Patients were informed about the iVR study through posters displayed within the cardiology unit, as well as through discussions with cardiologists and nurses.

### Immersive virtual reality technology

During all eight CR sessions, participants used an iVR headset, specifically the PICO 4®, paired with the TREADMILL XR® software. The latest generation of iVR technology was employed, offering high computational power, self-contained advanced software, and superior optical components. This setup delivered low latency, a wide field of view, and a significantly enhanced iVR experience. The PICO 4® was selected for its advanced features, ensuring high-quality performance. Participants experienced immersive, natural environments with 4 K video resolution and 360° spherical video content. To enhance the immersive experience, it is also important to stimulate the auditory sensory modality (29, 30). The TREADMILL XR® app was chosen for its realistic content and sounds, as interacting with such environments has been shown to effectively divert attention, providing a powerful distraction (31).

### Intervention

During the 30-min CR sessions, iVR was administered with the assistance of a healthcare professional and a cardiologist specialized in CR, both of whom were trained in iVR. Participants had the freedom to move their eyes and head, adjust the volume to their liking, and stop the iVR session at any point for any reason. Ten minutes before the first session, a healthcare professional trained in iVR provided instructions and demonstrations on how to use the technology, ensuring the patient was comfortable and proficient in operating the virtual equipment. Additionally, they offered continuous support throughout and after each session, promptly addressing any issues that arose. For safety, the healthcare professional monitored the patient’s iVR experience via a tablet and had the ability to stop the VR content or halt the treadmill if necessary. To monitor any adverse events related to the intervention, potential virtual reality sickness was assessed. This condition causes symptoms such as nausea, dizziness, fatigue, and eye-related issues. The Virtual Reality Symptom Questionnaire was used to evaluate 23 symptoms (12 related to non-ocular issues and 11 to ocular ones) on a 7-point scale (32). Symptoms were considered minimal if their severity was below 20% (33).

### Data collection

Data were collected from September 1st, 2024, to January 25th, 2025, in the cardiology unit of a public hospital in Italy. Semi-structured interviews were conducted face-to-face in a quiet, private space within the cardiology rehabilitation area, with only the participant and the researcher present, after the completion of the CR sessions with iVR. All participants completed the eight iVR sessions without interruptions, except for one patient who discontinued the use of iVR after the second session due to mild cybersickness and continued the rehabilitation sessions without iVR. The interviews were conducted with all patients at the end of the eight sessions. The interviews were carried out by two female nurse researchers trained in qualitative research: one with a PhD and the other with a master’s degree. The interviewers were also experts in iVR applications and in conducting research on digital health involving new technologies such as iVR. The interviewers did not know the participants. The interviews were conducted in a permissive, non-judgmental manner, allowing participants to speak freely and ensuring they had ample time to share their experiences. The interviews lasted an average of 20 min, were audio-recorded, and then transcribed verbatim by a research assistant. A researcher checked the transcriptions for accuracy and completeness. An interview guide, developed by the research team based on a literature review, including open-ended questions about the participants’ experiences with iVR, was used (Table 1). The participants were invited to describe their experience with iVR during CR, share which aspects of the experience impressed them the most, explain what they perceived as positive and negative aspects, and identify areas that could be improved. Additionally, they were asked to describe how they perceived the passage of time during the rehabilitation session. Only one interview was conducted with each participant. The transcripts were not returned to the participants for feedback or revisions.

**Table 1:** Interview guide on patients’ experience with immersive virtual reality.

Categories	Questions
Introductory question	Can you tell me something about your experience with virtual reality during cardiac rehabilitation?
Transition questions	Which aspects of the experience impressed you the most?
Key questions	What do you think might have been the positive aspects, the negative ones, and those that could be improved?
Key questions	How did you perceive the passage of time during the rehabilitation session?
Ending question	Do you have any other considerations to share?

### Data analysis

The transcriptions were analyzed using inductive content analysis, following the approach outlined by Graneheim and Lundman (25). The analysis was conducted manually, without the use of specific qualitative data analysis software. To enhance reliability, two researchers experienced in qualitative analysis independently coded the data, then compared and discussed their findings to reach consensus, under the oversight of two faculty members who are experts in the field. This double-coding approach contributed to enhancing the consistency and credibility of the results. Initially, the transcriptions were reviewed multiple times to grasp the overall meaning and ensure a proper understanding. Relevant words or phrases, referred to as meaning units, were then identified. These were compared and coded based on their similarities. The codes were subsequently grouped into subcategories and categories according to common features and differences.

### Rigor

To ensure the study’s rigor, the criteria of credibility, transferability, dependability, and confirmability, as outlined by Lincoln and Guba, were followed (34). Credibility was upheld by selecting an appropriate data collection method and gathering sufficient data. Transferability was supported by providing detailed descriptions of the participants’ socio-demographic characteristics, cardiovascular risk factors, lifestyle habits, and clinical profiles, as well as a description of the context, including how the rehabilitation was carried out in terms of both quantity and effort achieved by the participants, a detailed explanation of the technology used, and the healthcare professionals involved. This allowed readers to assess the applicability of the findings to other settings. Dependability was ensured through a well-documented, traceable, and logically structured research process. Finally, confirmability was guaranteed through consistency between the interpretations, findings, conclusions, and the data collected.

## Results

### Participant characteristics

Data saturation was reached after interviewing 22 participants. These participants were all Italians with a mean age of 68 years, 81% of whom were men. The majority were married (50%). In terms of education, 45% had completed high school, and 45% were retired. Cardiovascular risk factors included arterial hypertension (68%), hypercholesterolemia (45%), and moderate smoking and alcohol consumption. Only 32% of participants were physically active, and the average BMI (Body Mass Index) was 28.35. All patients were diagnosed with HF with a reduced ejection fraction, ranging from 23 to 39%, and the majority were functionally classified as NYHA (New York Heart Association) class II (59%). Chronic coronary syndrome was common, with 32% having undergone PTCA (percutaneous transluminal coronary angioplasty) and drung-eluting stent implantation in the past year. Further details regarding participants’ characteristics are presented in Table 2.

**Table 2:** Participant characteristics.

Characteristics	Classification	*n*	%
Sex	Female	4	18.00
	Male	18	81.00
Age (years)	Mean (SD)	68 (8)	
Nationality	Italian	22	100
Marital status	Single	3	13.63
	Married	11	50.00
	Living as married	4	18.18
	Divorced	2	9.09
	Widow/widower	2	9.09
Education level	Primary school	3	13.64
	Middle school	6	27.27
	High school	10	45.45
	Graduate	3	13.64
Occupation	Retired	10	45.45
	Unemployed	2	9.09
	Employed	10	45.45
Sudden cardiac death in second-degree relatives	Yes	1	4.55
	No	21	95.45
Early MI in 1st-degree relatives (<60 years old)	Yes	8	36.36
	No	14	63.64
History of peripheral artery disease	Yes	3	13.64
	No	19	86.36
History of cerebrovascular disease	No	22	100
NYHA Class	I	6	27.27
	II	13	59.09
	III	3	13.64
	IV	0	0
Left Ventricular Ejection Fraction (%)	Min	23	
	Max	39	
	Mean	31	
	Median	32	
History of PTCA/Stent in the last year	Yes	7	31.82
	No	15	68.18
Arterial hypertension	Yes	15	68.18
	No	7	31.82
Hypercholesterolemia	Yes	10	45.45
	No	12	54.55
Tobacco use	Never	12	54.55
	Previous	7	31.82
	Actual	3	13.64
Alcohol use (> 2 alcohol units/day)	Yes	7	31.82
	No	15	68.18
BMI	Min	22	
	Max	34.7	
	Mean	28.35	
	Median	29.1	
Habitual physical activity	Yes	7	31.82
	No	15	68.18

SD = Standard Deviation; Min = Minimum; Max = Maximum; PTCA = Percutaneous Transluminal Coronary Angioplasty; MI = Myocardial Infarction; NYHA = New York Heart Association; BMI = Body Mass Index.

### Findings

From the analysis of the interviews, nine subcategories grouped into four main categories were extracted: (1) cognitive and emotional benefits of iVR; (2) impact on rehabilitation; (3) customizable intervention, (4) negative aspects of the iVR. The main categories and subcategories, along with excerpts from the interviews, are presented in the paragraphs below and illustrated in Table 3. Participants are identified by alphanumerical codes assigned in the RCT, which are included in parentheses with each excerpt.

**Table 3:** Qualitative findings.

	Subcategories	Codes	Condensed meaning units
Cognitive and emotional benefits of iVR	Acceptability and enrichment of the rehabilitation experience	Acceptability	They lived a positive experience
		Sense of discovery	They could see unknown places They described the experience as a journey
	Positive emotions	Perception of emotional well-being	They perceived serenity, calmness, pleasure
		Perception of freedom	They felt free
		Reduction of negative thoughts	They did not think about illness
		Relief from anxiety	They were distracted from anxiety
		Relaxation	The iVR helped them relax
		Enjoyable	They enjoyed themselves
	Cognitive engagement	Immersion in the scenarios	They were full immersed in the scenarios
		Distraction	They felt removed from the clinical setting.
		Perception of time passing quickly	They felt the time passed faster
		Motivation	They were motivated to continue
Impact on rehabilitation	Physical engagement	Encouragment to movement, reduction of inactivity	They were encouraged to actively participate in rehabilitation
	Perceived effort	Distraction from physical effort	They felt less focused on the effort required
Customizable intervention with beautiful images	Variety of scenarios	Appreciation for the different natural scenarios	They liked the scenarios with woods, animals, mountains, beaches and lakes
	Quality and beauty of the videos	Appreciation of the quality of the images in the videos	Patients liked the colours, the music, and the beauty of the videos
Negative aspects of iVR intervention	Discomfort with the equipment	Heavy Headset	The headset was perceived as heavy
		Issues with image focus	Some images were perceived as blurry
		Incomplete isolation from external sounds	Patients could not isolate completely because they were hearing external sounds.
		Unfamiliarity with technology	Inexperience in using the devices
	Negative feelings toward the iVR devices	Frustration in using the devices	Irritation for not knowing how to use the devices

#### Cognitive and emotional benefits of iVR

This main category cognitive and emotional benefits of iVR included three subcategories: “acceptability and enrichment of the rehabilitation experience,” “positive emotions,” and “cognitive engagement.” It describes how new experiences with iVR have enriched participants’ understanding of new technologies, provided pleasant experiences of new places and scenarios, positively impacting their emotional well-being, and offered cognitive benefits, such as distraction from negative hospital surroundings and a new perception of time.

##### Acceptability and enrichment of the rehabilitation experience

Many participants reported an improvement in the acceptability of the rehabilitation experience due to the use of this innovative technology. They noted: “*The experience was absolutely positive, exceeding all expectations.*” (ID 6); “… *It does something that makes it easier, more acceptable, it helps…*” (ID 24); “… *Great for avoiding the boredom of repetitive exercise…*” (ID 19); “*… I think rehabilitation is a very good thing to do in this way…*” (ID 20); “… *It was as if I had gone for a walk*…” (ID 29); “*I would recommend it to other people*” (ID 10). Participants’ knowledge and experiences were further enriched by encountering unfamiliar and previously unknown places depicted in the scenarios. For instance, participants reported: *“*… *I saw places I had never been to before”* (ID 4); “… *It was interesting to see places I did not know*… *making the session feel more like an exploration*…” (ID 11); “*It felt like I was on a journey.”* (ID 33).

##### Positive emotions

iVR had a significant positive impact on participants’ emotional well-being. Many described feelings of enjoyment, relaxation, and ease. For example, patients shared: “*It was positive, it could not have been better*.” (ID 2); “*I felt better afterward*” (ID 2); “… *Completing a pleasant and effortless recovery*…” (ID 19); “… *It was extremely enjoyable*…” (ID 20); “… *It did me good*…” (ID 29); “*Being in the forest gave me serenity and calm*” (ID 23). Additionally, many participants experienced a sense of freedom, feeling liberated from the emotional burden of their illness. As some noted: “*You feel free, as if you are in the middle of nature*” (ID 26); “*With the VR headset, I did not think about my health problems*…” (ID 16); “… I *had no worries*…” (ID 29); “…*I went on calmly*…” (ID 29). Another significant aspect was that iVR provided relief and distraction from anxiety. A participant shared: *““With the headset, I distracted myself from the anxiety I always felt when doing rehabilitation”* (ID 30). Furthermore, iVR helped participants to relax during CR positively influencing their mind–body connection. As they noted: “… *It made me feel very relaxed*…” (ID 24); *“*… *it is able to relax the people participating in the rehabilitation… (ID 4);* “…*Watching the videos relaxes you, it’s a good way to do the activity without spending the whole session staring at the wall*” (ID 4). Patients enjoyed themselves and found the iVR experience highly engaging, showing deep interest in the scenarios: *“*… *the aspects I appreciated the most was the* var*iety of scenarios offered*…” (ID 6); *“The experience was quite enjoyable*” (ID 8); *“This experience was very interesting for me*…*”* (ID 33).

##### Cognitive engagement

Participants perceived iVR as a distraction from their negative surroundings as they were transported to another place, away from the cardiological ward. They felt fully immersed in the scenarios: *“It was also a bit of fun, I did not even realize it*…*”* (ID 18)*; “… you feel so immersed in the video that you even perceive the dangers of the scenario”* (ID 15). The immersion was closely linked to distraction, which lead to the shift of attention from the negative external environment toward the beneficial internal cognitive experience produced by the iVR: “*When you follow the video, you disconnect*…*” (ID 8);* “… *It’s a system that tends to remove that hospital-like aspect*…” (ID 24).

Moreover, iVR altered participants’ perception of time with time passing more quickly: *“*… *Virtual reality makes you not think about the passing of time*…*”* (ID 1); *“it felt like time passed quickly during all the sessions*.” (ID 6); “*in some cases, it was even too fast”* (ID 1); *“*… *Time flies without you realizing it*…” (ID 18). Moreover, iVR has increased motivation in rehabilitation: “*it feels almost as though you are pedalling with less effort because you are psychologically encouraged to follow the music and everything around you* “(ID 23); “*Having results could motivate the patient more, rather than undergoing treatment in a passive way*” (ID 24); *“I think it helped me stay motivated and made the sessions feel less like a chore*” (ID 33).

#### Impact on rehabilitation

This main category encompasses two subcategories: “physical engagement” and “perceived effort.” It describes how new experiences with iVR have fostered greater involvement in rehabilitation activities and changed participants’ perceptions of the perceived effort.

##### Physical engagement

iVR encouraged participants to engage with rehabilitation equipment like treadmills and exercise bikes, increasing their involvement in the process and even inspiring some to recreate similar setups at home to maintain their progress. As one participant stated, “*… and I started using the treadmill. I’m not saying I do it every day at home now, but I do use it…*” (ID 1). Another remarked, “*I even thought about possibly seeing if it’s possible to buy and set up something similar at home, also because it’s very boring to just sit still*” (ID 20), highlighting the potential for the experience to spark further interest in physical activity. One more participant expressed, “*I would like to continue doing rehabilitation if possible*.” (ID 29), emphasizing the positive impact on continued engagement.

##### Perceived effort

The iVR experience helped reduce participants’ awareness of the physical effort involved in rehabilitation. The immersive environment distracted them from the exertion required for the exercises. One participant shared, *“It allowed me to focus more on the experience rather than the physical effort involved”* (ID 18), while another noted, *“There’s a bit of music giving rhythm, it almost feels like cycling takes less effort”* (ID 20). Additionally, one participant described *“Effortless recovery was a truly impressive aspect”* (ID 19), highlighting how the virtual reality experience made the rehabilitation process feel less demanding.

##### Customizable intervention

This main category included two subcategories: “variety of scenarios” and “quality and beauty of the videos.” It illustrated the participants’ appreciation for the several scenarios available to choose from, and their perceptions of the quality and beauty of the images, scenes, colors, sounds, and music.

##### Variety of scenarios

Participants found that natural scenarios provided an enjoyable experience. Some patient expressed a sense of enjoyment from the variety of scenarios with the nature: “*Each session felt unique, and the playful nature of the experience made the entire process much more enjoyable.”* (ID 6); *“*… *It gives the feeling of being in nature*…*”* (ID 10). Other participants were positively affected by the presence of animals in the scenarios, feeling closeness to them: *“I found some forest animals right next to me, how strange” (ID 23)*. Other patients appreciated the mountain scenarios: *“*… *It somewhat depends a lot on the video, like the mountain”* (ID 8); “… *I see the mountains and think how wonderful it would be to reach the summit*” (ID 10), woods scenarios; “I felt good when I was cycling in the woods…” (ID 30); lake scenarios “… *The lakes were really beautiful”* (ID 11); beach scenarios” … *these are very pleasant things: a beach*…” (ID 20).

##### Quality and beauty of the videos

Participants appreciated the colors, the music, and the beauty of the videos, which featured realistic, convincing images and sounds: *“Following the music, following everything around you, these are very pleasant things: a beach, the greenery, the mountains, the lakes*… *all in all, very enjoyable*…*” (ID 20);* “… *I was impressed by the landscapes in the headset*…” (ID 29); “*While I was cycling, I found myself captivated by the beautiful colours of nature*…*.”* (ID 2);” *Another positive element was the music that gave me rhythm.*” (ID 11); “… *I see a reality that I like*…*”* (ID 10); “… *I had the impression that the movements in the videos were real*” (ID 10).

#### Negative aspects of iVR

This main category included two subcategories: ‘discomfort with the equipment’ and ‘negative feelings toward the iVR devices.’ These subcategories describe the negative experiences participants had with iVR technology.

##### Discomfort with the equipment

Some participants reported some discomfort with the equipment iVR headset: “*The headset sometimes bothered me a bit” (ID 16).* Other patients noted discomfort with image focus: “… *it’s often out of focus, never completely sharp*…” (ID 20). Other issues included a lack of isolation from external sounds during the iVR session: “*Sometimes I could hear the sounds in the room” (ID 23).* Inexperience in using the iVR devices was also reported: *“I did not know Virtual Reality, and this caused me some difficulty at the beginning*…*” (ID 30).* Finally, some participants reported difficulty in the following framing changes: *“The mountain made me feel dizzy*…*” (ID 24);* “*I felt like I was falling into the lake*” (ID 15).

##### Negative feelings about the inability to use the iVR device

Some patients reported irritation for not knowing how to use the devices: *“The simplicity of the installation could be improved*…*” (ID 24);* “…*could be improved by using more suitable devices that allow interaction.”* (ID 35).

## Discussion

This qualitative study, embedded within an RCT, aimed to explore patients’ experiences with iVR during eight CR sessions. The results highlighted that the use of iVR during CR had positive impacts on various aspects, although some negative experiences related to the technological aspects were also noted. Firstly, iVR offered significant cognitive and emotional benefits to the participants.

Patients reported an enhanced sense of acceptability and enrichment in their rehabilitation experience, as they explored new and previously unseen scenarios. This aspect helped reduce boredom and fostered a positive intrinsic motivation toward the rehabilitation process, making the experience less monotonous and more enjoyable. Additionally, the immersive environment offered a significant distraction from the hospital setting, which was perceived as negative and stressful. This distraction also helped improve anxiety management, offering a moment of relaxation that positively impacted the patients’ psychological well-being. Furthermore, iVR emerged as a valuable tool for enhancing patients’ participation in rehabilitation by reducing the perception of effort, making exercises feel less strenuous and more engaging. Another positive aspect was the ability to customize the rehabilitation experience through the selection of various scenarios, which contributed to making each session unique and enhancing patient-centered care (35). The diverse natural landscapes, forests, lakes, mountains and the interaction with animals had a profound emotional impact on the participants, making the experience both stimulating and enjoyable (31). This aligns with the literature, which highlights that the sense of presence in iVR is heightened when it elicits genuine cognitive, emotional, and behavioral responses while also allowing participants to construct their own narrative of the experience (36). Furthermore, natural scenarios are known to enhance well-being (37). Their immersive qualities, enriched by colors, sounds, and variety, promote relaxation and emotional well-being, helping to reduce anxiety (38).

Our study also revealed some physical benefits that were indirectly observed during the qualitative interviews. Participants reported a greater engagement in physical activity and a reduced perception of exertion, which aligns with previous findings highlighting that immersive experiences can influence physical responses (39). However, to quantify these effects and confirm them, quantitative studies are needed.

Our study also revealed negative aspects of the iVR intervention, such as discomfort with the equipment and negative feelings related to the inexperience in using the iVR devices. As in previous studies, patients reported discomfort with the iVR headset and expressed the need for easier adjustments (36, 40). Although the head-mounted displays used during the study were among the best available at the time, they presented several technological issues, such as visor heaviness, image blurriness and lack of isolation from external sounds. These issues can be addressed by the latest generation of iVR devices, which will be lighter, more comfortable to wear, and easier to manage even during movement and physical activity, with more precise and sophisticated automatic focus systems. In any case, since some of the reported focusing issues may have been caused by patients’ difficulty in managing ocular variables, likely due to a lack of experience with the iVR headset or inadequate understanding of the instructions provided by healthcare professionals, it is advisable to dedicate 10 min to a familiarization phase on the first day of VR use to help patients acclimate (41).

Our findings are consistent with those of Alves da Cruz et al. (42), who reported that patients with cardiovascular diseases experienced high levels of satisfaction and perceived both physical and mental benefits from their involvement in iVR-based rehabilitation, as highlighted in their qualitative study involving 15 participants. However, our study places greater emphasis on the positive emotional effects and the personalization of the rehabilitation experience through diverse scenarios. In contrast, Alves et al.’s study focuses more on the social interaction benefits resulting from the gamified features of iVR, such as competition and game mechanics. A notable difference between the two studies lies in the role of motivation. In our study, motivation was identified as a crucial factor for the success of iVR-based rehabilitation, which aligns with other studies showing that iVR enhances motivation (43), whereas in Alves et al.’s study, motivation was not found to be associated with the iVR intervention. Patients in their study reported that their motivation to engage in physical exercise was primarily driven by the awareness of its health benefits, rather than by the iVR experience itself (42). Both studies agree on the importance of familiarizing patients with technology and addressing the practical challenges related to VR use, underscoring the necessity for gradual adaptation and proper supervision to ensure the successful integration of iVR in rehabilitation (42). Another theme that did not emerge in Alves et al.’s study is the effect of iVR on the perception of the passage of time. In contrast, this theme was prominently featured in the study by Burrai et al. (44), where the mechanisms influencing time perception were found to be directly proportional. Specifically, as the depth of attention, distraction, and presence triggered by virtual scenarios increased or decreased, so too did the perceived speed at which time passed for the patient. The ability of iVR to immerse, distract, enjoy, and generate a strong sense of presence within the virtual environment leads patients into a state in which time is perceived as passing more quickly (44, 45). This state is considered crucial for alleviating the psychological burden often associated with repetitive and fatiguing rehabilitation exercises (46), while also fostering positive psychological responses such as enhanced intrinsic motivation, improved mood, and increased self-efficacy, factors that collectively contribute to sustained adherence to rehabilitation over time (7, 8).

These findings are consistent with existing quantitative studies that have investigated the psychological aspects of using sVR during CR. Specifically, they align with the systematic review and meta-analysis by Bashir et al. (17), which demonstrated a significant reduction in anxiety levels among the patients included in the study. Similarly, the results align with those of the study by Gulik et al. (47), where patients also reported enjoying their sVR experience. Additionally, Morgan et al. (48) reported a higher overall level of satisfaction among participants, which further supports our findings. Finally, the research by Chang et al. (49) also showed that iVR led to greater satisfaction among patients, reinforcing the positive effects of sVR. The impact of iVR on physical and psychological symptoms in CR is still underexplored. However, it is important to consider the potential differences between sVR and iVR technologies, as these influence patients’ experiences in distinct ways. iVR offers a stronger sense of presence and engagement, enhancing distraction from the clinical environment and reducing perceived effort, while sVR may be more accessible, especially for those less familiar with technology, but it does not guarantee complete isolation from the patient’s environment, a crucial aspect for activating the mediators that lead to actual improvements in outcomes (7). A clearer understanding of these differences may guide the selection of VR tools best suited to the specific needs and contexts of patients, thus improving the personalization and effectiveness of rehabilitation programs.

Several positive aspects can be highlighted in relation to our study. Firstly, the patient experience with iVR was structured around a total of eight sessions, which allowed for the inclusion of long-term psychological effects. Additionally, another positive aspect is that the rehabilitation duration was set at 30 min, in accordance with safety recommendations that suggest limiting the duration to this time to reduce the risk of cybersickness. In fact, this phenomenon occurred in only 2% of the sessions, observed in a single patient who experienced mild headache symptoms. One limitation of this study is its monocentric design. Furthermore, the sample consists solely of Italian participants, predominantly men, with a mean age of 68 years. As a result, the findings may not be generalized to more diverse populations, particularly those with varying ages, ethnic backgrounds, or geographic locations. Another limitation of our study concerns the composition of the sample, which includes only patients who completed all eight sessions with iVR, except for one who discontinued due to mild cybersickness. Since adherence to rehabilitation is a known issue, especially for vulnerable groups such as women, the elderly, and individuals with logistical and social challenges, the results may not be generalizable to those who have difficulties completing the treatment. This aspect should be considered when interpreting the results, as it may limit the transferability of the conclusions.

### Implications for practice and further research

The study highlights the potential of iVR as an effective tool for enhancing patient cognitive, emotional, and physical benefits. It is well accepted by patients, easy to use for healthcare professionals, and associated with minimal side effects. To further reduce the risk of cybersickness, it is recommended to dedicate 10 min to a familiarization phase on the first day of iVR use to help patients acclimate (41). It is also essential that healthcare professionals using iVR receive adequate training both in the use of the technology and in managing potential discomfort, such as focus adjustment requests, in order to ensure an optimal patient experience. Moreover, they should be able to recommend the most appropriate scenarios based on the rehabilitation goals and clinical context.

Future research should integrate both qualitative and quantitative approaches to evaluate the impact of iVR on rehabilitation adherence, clinical outcomes, and cost-effectiveness. To optimize the use of iVR and support its effective integration into clinical practice, it will be important to address the limitations of this study by including patients at risk of low adherence, expanding sample diversity through multicenter studies, and further exploring the differences between iVR and sVR in relation to patients’ specific needs.

## Conclusion

iVR is an innovative intervention that can enhance the humanization of care and promote a patient-centered approach for individuals undergoing CR. Our data suggests that iVR may be an effective and customizable intervention offering cognitive, emotional, and physical benefits. The ability to personalize the experience, combined with its positive effects on time perception, motivation, enjoyment, effort perception, and anxiety management, highlights iVR’s potential in rehabilitation. However, certain technological challenges, such as discomfort with the equipment and difficulty adapting to the technology, need to be addressed to optimize the user experience. To enhance patients’ experiences, more emphasis should be placed on familiarizing them with iVR, dedicating additional time to training, and using the latest technology.

## Data Availability

The raw data supporting the conclusions of this article will be made available by the authors, without undue reservation.
